# Study on the promotion of lymphocytes in patients with COVID-19 by broad-spectrum chemokine receptor inhibitor vMIP-II and its Mechanism of signal transmission in vitro

**DOI:** 10.1038/s41392-021-00516-4

**Published:** 2021-03-02

**Authors:** Shiyu Li, Shuting Liu, Zhenyou Jiang, Lixia Feng, Yong Gao, Youyu Chen, Anding Xu, Wenhua Huang, Nuofu Zhang, Hanxiao Sun

**Affiliations:** 1 Institute of Genomic Medicine, College of Pharmacy, Jinan University, Guangzhou, China; 2 Department of Anatomy, School of Basic Medical Sciences, Southern Medical University, Guangzhou, China; 3 Departments of Microbiology and Immunology, Medical College, Jinan University, Guangzhou, China; 4 Nanyang People’s Hospital, Henan, China; 5 Union Hospital, Tongji Medical College, Huazhong University of Science and Technology, Wuhan, China; 6 Guangzhou Overseas Chinese Hospital, Guangzhou, China; 7 Institute of Respiratory Diseases, Guangzhou, China

**Keywords:** Immunological disorders, Adaptive immunity

**Dear Editor**,

COVID-19 cases showed a significant decrease in the number of peripheral CD4^+^ and CD8^+^ T cells accompanied by overactivation of cells.^1^ HHV8-derived viral macrophage inflammatory protein vMIP-II is the only broad-spectrum chemokine receptor inhibitor that binds to three subtribes of chemokine receptors, which can significantly increase the specific cell immune response in infected organisms and have an effect of reducing plasma viral load on the viremia period.^2–4^ Here we report the effect of vMIP-II in COVID-19.

This study included 10 patients and 35 uninfected patients from Wuhan Union Medical College Hospital and Nanyang First People’s Hospital. Lung CT scans before and 1 week after vMIP-II treatment showed the ground glass lesions and white areas of the lungs were significantly alleviated, clinical symptoms were significantly reduced. The examination of convalescent blood samples showed a significant increase in the total number and proportion of lymphocytes (Supplementary Table S1 and Fig. 1a). The median time for the virus to turn negative was 16 (SD ± 2.2) days, which was significantly shorter than that in the conventional treatment group (22 days, SD ± 3.5) (Supplementary Table S2).

**Fig. 1:**
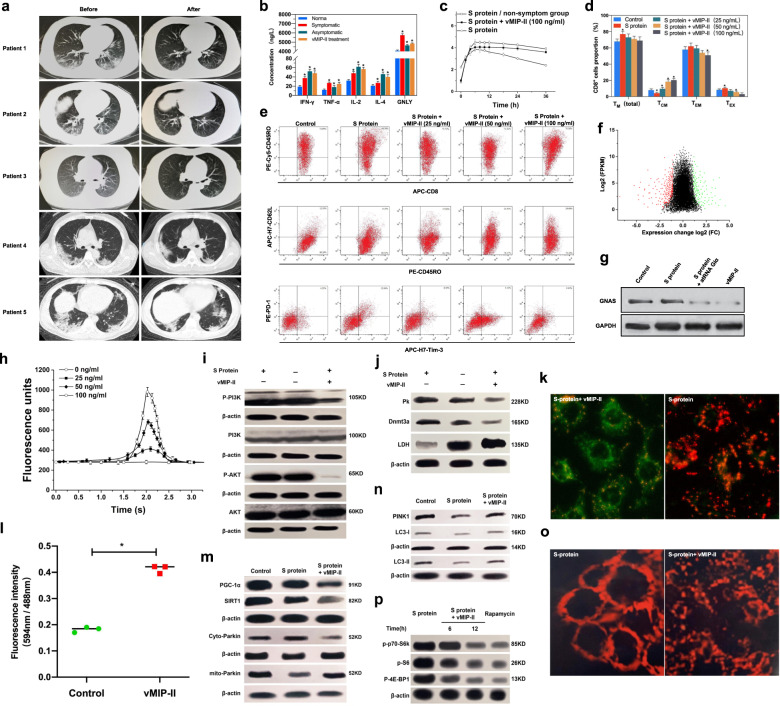
Chemokine receptor inhibitor vMIP-II promotes the conversion of effector CD8^+^ T into T_CM_ cells in the reconstitution of cellular immunity in patients with COVID-19. **a** Reduction of lesions of lung CT treated by vMIP-II. Five common patients with SARS-CoV-2 virus were scanned by lung CT before and 1 week after vMIP-II treatment. The ground glass lesions and white areas of the lungs were significantly alleviated after 1-week treatment. **b** Cytokine level of different groups of PBMCs stimulated by S protein in convalescent patients after 1 week of being negative. Different groups of separated PBMCs were added with S protein (100 ng/mL S1 + S2, w/w = 2:1) and detected by cytokine kits, *p value < 0.05. **c** Proliferation of PMBCs stimulated by S protein in convalescent groups after 1 week of being negative. Compared with the common symptomatic group, vMIP-II group and non-symptomatic group, *p-value < 0.05 (*n* = 5). **d** Flow cytometry detection of memory CD8^+^ T cells. Different subgroups of memory CD8^+^ T cells were distinguished by CD45RA and CD62L. The third line was the detection of inhibitory molecules on the surface of CD8^+^ T cells. The depleted CD8^+^ T cells (T_EX_) were distinguished by the highly expressed inhibitory molecules PD-1 and Tim-3. **e** Proportion and distribution of CD8^+^ T-cell subgroups in different vMIP-II to S protein. Compared with control group, * *p* value <0.05. (*n* = 3). **f** MA map of differentially expressed genes. Gene differential expression analysis on effector CD8^+^ T cells sample of vMIP-II treament group and S protein group was performed by DESeq. **g** Western blotting detected that vMIP-II reduced the G protein expression of CD8^+^ T cells. In the presence of Gi α antisense oligodeoxynucleotides, the reduction of G protein induced by vMIP-II is more obvious. **h** Compared with the control group, vMIP-II, significantly inhibited the rapid influx of calcium, and the *p* value of the S protein and vMIP-II groups was <0.01. **i**, **j** The effect of vMIP-II on the phosphorylation of PK, LDH, Dnmt3a, PI3K, and Akt. Under S protein stimulation, CD8^+^ T cells were incubated with vMIP-II or without vMIP-II. The results showed that in the presence of vMIP-II, the phosphorylation level was significantly reduced. **k**, **l**. vMIP-II reduced the mitochondrial membrane potential of CD8^+^ T cells. The left side is the vMIP-II treatment group, and the right side is the control group. Compared with the control group, the intensity of the fluorescent color was reduced, and the *p value was <0.01. **m**, **n**. The effect of vMIP-II, on mitochondrial proliferation genes SIRT1, PGC-1ɑ, and autophagy genes LC3, PINK1, Parkin genes. Under S protein stimulation, CD8^+^ T cells were incubated with vMIP-II or without vMIP-II . The results showed that in the presence of vMIP-II, mitochondrial proliferation-related genes were suppressed and autophagy-related genes increased. **o** The effect of vMIP-II on mitochondrial network structure. The left side is the S protein control group, and the right side is the vMIP-II treatment group. It can be seen that fragmented mitochondria appeared after vMIP-II treatment, and the mitochondrial network was destroyed, 400×, *p value < 0.01. **p** The effect of vMIP-II on downstream proteins of mTOR pathway. Under S protein stimulation, the S protein CD8^+^ T cells were incubated with vMIP-II for 6 h or 12 h. The results showed that in the presence of vMIP-II, the downstream proteins of the mTOR pathway were inhibited at 6 h, and the inhibition was more obvious at 12 h

In order to explore the mechanism of vMIP-II on the efficacy of COVID-19, we examined the responsiveness of blood PBMCs to S protein in recovering patients. First, we perform the cloning and expression of S protein (Supplementary Fig. S1, 2, 9). S protein had antigenic characteristics to PBMCs. The classification of CD8^+^ T-cell subgroups based on the PBMCs of convalescent patients showed that the ratios of T_CM_ cells in the vMIP-II treatment and asymptomatic infection groups were significantly higher than that in the symptomatic infection group, although there was no significant difference in the total number of lymphocytes (Supplementary Table S3, Supplementary Fig. S4). There were also differences in the secretion type of PBMC cytokines and the ability to stimulate the proliferation of PBMCs in different groups treated with S protein (Fig. 1b, c). There was consistency between the vMIP-II treatment group and the asymptomatic infection group.

CD8^+^ T lymphocyte subgroup analysis on normal PBMCs treated with S protein and vMIP-II was performed (Supplementary Table S3, Fig. 1d, e). In the three vMIP-II treatment groups, there was no significant change in the total number of the memory CD8^+^ T cells, but the number of CD8^+^ central memory (T_CM_) cells increased significantly with the dose. In vMIP-II group, the levels of Th1 cytokines (IFN-γ, IL-2) were significantly increased, but the levels of TNF-α and IL-4 were significantly decreased. There were no significant differences in granulysin levels among the groups (Supplementary Fig. S4).

We applied RNA-seq to detect differential CD8^+^ T cell gene expression in the vMIP-II treatment group (100 ng/mL S protein + 100 ng/mL vMIP-II) and the S protein group (100 ng/mL S protein) (Fig. 1f). A total of 97 significantly differentially expressed genes were screened by the standard. GO ontological analysis and KEGG pathway analysis showed that phosphorylation pathway, TCA cycle and apoptosis regulation were the most abundant GO terms for the three differentially expressed genes (Supplementary Tables S5, S6, S7 and Supplementary Fig. S5a). We selected the differentially expressed genes with fold differences > 3. Ultimately, GNAT1, PI3K, p38mapk, AKT, TSC1, PINK1, LC-3, BCL-2, FAS, CXCR4, CX3CR1, CCR5, CCR7, PK, LDH, and SIRT1 were identified as key target genes for our subsequent research. We used qRT-PCR to verify the differentially expressed genes of these 16 genes (Supplementary Fig. S5b).

The results of gene sequencing and qRT-PCR showed that vMIP-II affected the α subunit of Gi protein in effector CD8^+^ T cells (the expression of GNAT1 gene was decreased). In response to this, we also carried out Gi α antisense oligodeoxynucleotides assay. Compared with S protein group, the expression of Gi α in effector CD8^+^ T cells treated with vMIP-II was significantly inhibited (Fig. 1g). vMIP-II affects rapid intracellular calcium ion concentration and induces calcium release from intracellular calcium ion pools (Fig. 1h and Supplementary Fig. S6). In the presence of vMIP-II the phosphorylation levels of PI3K and Akt in the Giα protein-coupled phosphorylation pathway were significantly decreased and the expression level of Dnmt3a, PK protein was also decreased while the protein expression level of LDH was significantly increased (Fig. 1i, j). This suggests that the metabolic mode of ATP in cells has changed.

The effect on cell function was further observed, and the level of cell ROS was detected, the green fluorescence of CD8^+^ T effector cells treated with vMIP-II was stronger than that in the S protein group (Supplementary Fig. S7). It indicates that mitochondrial damage occurred. To detect the effect of vMIP-II on mitochondrial function in CD8^+^ T cells, we detected changes in mitochondrial membrane potential in S protein treated CD8^+^ T cells (Fig. 1k, l), the results showed that the mitochondrial membrane potential decreased. Because the cellular mitochondrial network is affected by the balance of mito proliferation genes and autophagy genes. We detected and found that after vMIP-II treatment, the proliferation gene SIRT1, PGC-1α of CD8^+^ T effector cell mitochondria were significantly inhibited and mitochondrial autophagy gene LC3-II, LC3-I, PINK1, Parkin were activated. In addition, we also detected that the mitochondrial network was broken (Fig. 1m, n, p). The role of vMIP-II in effector CD8^+^ T cells is to transform energy ATP metabolism from oxidative phosphorylation to glycolysis by weakening the function and network of mitochondria, which is a characteristic of cell dry transformation.

We further explore the causes of mitochondrial dysfunction. We have found that the inhibition of the phosphorylation pathway will cause the expression of PI3K, AKT to decrease, that is, affecting the PI3K-AKT-mTOR pathway. In order to verify that mitochondrial function is impaired by inhibiting the downstream target protein of mTOR, we used the phosphorylation levels of the downstream target molecules S6K, S6, and 4E-BP1 of mTORC1 as indicators. Western blot analysis found that compared with S protein group CD8^+^ T cells, the phosphorylation levels of S6K, S6, and 4E-BP1 of the cells treated with vMIP-II decreased significantly at 6 h, and were strongly inhibited at 12 h, which was consistent with that of rapamycin group (Fig. 1o). These results confirm that the damage of mitochondrial function caused by vMIP-II is realized by inhibiting the downstream target protein of mTOR.

In summary, we observed that vMIP-II could significantly improve the lymphocyte decrease of COVID-19. vMIP-II inhibits multiple chemokine receptors and their related phosphorylation signaling pathways. The low level of pathway PI3K-AKT-mTOR reduces the network and function of mitochondria, to change cell energy metabolism to glycolysis, and also affects the activity of methylase Dnmt3a. The reduced phosphorylation leads to the transformation of effector CD8^+^ T cells into stem cells, producing more CD8^+^ T_CM_. This study shows that vMIP-II can reconstruct the cellular immune function lost in acute SARS-CoV-2 infection. The elucidation of the molecular mechanism of vMIP-II increasing CD8^+^ T_CM_ provides a new strategy for the treatment of COVID-19.

## Supplementary information

Supplementary Materials

instructions for the use of vMIP freeze-dry powder injection

Summary of clinical trial vMIP to COVID-9 in Union Hospital

Clinical Approval of vMIP for AIDS from National CDE
