# Supplementary material for: Study on the promotion of lymphocytes in patients with COVID-19 by broad-spectrum chemokine receptor inhibitor vMIP-II and its Mechanism of signal transmission in vitro
Source: Signal Transduct Target Ther. 2021 Mar 2;6:104. doi: 10.1038/s41392-021-00516-4 (PMC7921284; doi:10.1038/s41392-021-00516-4)
Supplement: Supplementary file 1 — Supplementary Materials [file 41392_2021_516_MOESM1_ESM.docx]

**Supplementary Materials for**

**Study on the** **promotion of lymphocytes in patients with COVID-19 by broad-spectrum chemokine receptor inhibitor vMIP-II and its Mechanism of signal transmission in vitro**

Shiyu Li^1,2*^, Shuting Liu^1*^, Zhenyou Jiang^3*^, Lixia Feng^4^, Yong Gao^5^, Youyu Chen^1^, Anding Xu^6^, Wenhua Huang^2✉^, Nuofu Zhang^7✉^, Hanxiao Sun^1✉^

^1^ Institute of Genomic Medicine, College of Pharmacy, Jinan University, Guangzhou, 510632, China.

^2^ Department of Anatomy, School of Basic Medical Sciences, Southern Medical University, Guangzhou, 510515, China.

^3^ Departments of Microbiology and Immunology, Medical College, Jinan University, Guangzhou, 510632, China.

^4^ Nanyang People's Hospital, Henan, 473002, China.

^5^ Union Hospital, Tongji Medical College, Huazhong University of Science and Technology, Wuhan, 430022, China.

^6^ Guangzhou Overseas Chinese Hospital, Guangzhou, 510632, China.

^7^ Institute of Respiratory Diseases, Guangzhou, 510030, China.

^*^ These authors contributed equally to be first author.

^✉^ These authors contributed equally to be Corresponding author,

**Wenhua Huang,**

E-mail: huangwenhua2009@sina.com;

**Nuofu Zhang,**

E-mail: nfzhanggird@163.com;

**Hanxiao Sun.**

E-mail: sunhx718@163.com

**This PDF file includes:**

Materials and Method

Fig. S1 Construction scheme of pEa-SARS-CoV-2-S1 and pEa-SARS-CoV-2-S2 plasmid.

Fig. S2 Clone, expression and antigenic activity of COVID-19 S protein.

Fig. S3 Analysis of T cell subgroups in PBMCs of convalescent patients after one week of being the virus negative.

Fig. S4 Effects of vMIP-II on the level of cytokines secretion of CD8^+^ T cells induced by S protein.

Fig. S5 Analysis of KEGG pathway of differentially expressed genes and its qRT-PCR verification.

Fig. S6 Measurement of vMIP-II-induced calcium release.

Fig. S7 The ROS level of CD8^+^ T cells.

Fig. S8 Patients lung CT.

Fig. S9 SDS-PAGE electrophoresis of S2 cloned.

Table S1 to S8

Materials and Method

**1.1 S protein antigen preparation**

The S-protein of SARS-CoV-2 Guangzhou strain occupies bases 21563-25384 of its genome, corresponding to 1273 amino acids, and encodes a protein of 141.2 kDa. Its cleavage activation site is YKTPPI, and it is cleaved into the S1 (25 kDa) and S2 (55 kDa) subunits.

Using His-tagged PQE-TriSystem (Invitrogen) as a plasmid, the SARS-CoV-2 S1 and S2 subunit genes were amplified and recombined to obtain pQE-His SARS-CoV-2-S1 and pQE-His SARS-CoV-2-S2. The construction scheme is shown in the Fig. S3. The plasmids were digested and sequenced for confirmation and transfected into Escherichia *coli* M15 for culture. The speed and ventilation was controlled during bacterial fermentation culture, and dissolved oxygen was set ≥ 40%. When bacterial optical density (OD) as measured at OD_600_ = 0.8, the inducer IPTG (1 mM) was added. After fermentation, we collected bacteria, then added inclusion body lysis buffer and centrifuged for 10 min. The collected inclusion body supernatant was passed through an affinity Ni column to obtain crudely purified S1 and S2 proteins. The processed protein was loaded onto a Superdex-75 molecular sieve and a DEAE column in sequence and eluted with an elution buffer. The eluted peaks were collected to obtain purified S1 and S2 proteins. Purity was determined by HPLC.

**1.2 Identification of S1 and S2 protein activity**

For the isolation of PBMCs, lymphocytes were isolated according to the conventional method of peripheral blood lymphocyte separation. The cell concentration in RPMI1640 medium was adjusted to 2.5 × 10^6^/mL, and 20 μL of 5 mg/mL MTT solution was added and incubated for 4 h. The supernatant was discarded, and 150 µL of DMSO was added to dissolve the precipitate. OD values were measured at 490 nm, and cell viability was calculated by the following formula: (%) = (OD treatment group/OD blank) × 100%. The experiment was performed in triplicate. Cells with viability > 90% were used in the following experiments.

After mixing the S1 and S2 proteins at a ratio of 2:1, the PBMCs were stimulated. Cells were seeded at 5 × 10^4^/well in 96-well plates and incubated with S protein for 12 h to examine changes in morphology. Samples were divided into the blank control group, S protein stimulation group (10, 50, and 250 ng/mL), and PHA (2 μg/mL) group. There were three replicates within each group.

**1.3 vMIP-II for the treatment of SARS-CoV-2 infection**

This research was approved by the Ethics Committee of Union Hospital, Tongji Hospital of Huazhong University of Science and Technology (No. 2020-0006), after obtaining informed consent and obtaining appropriate approval from the institutional review board, a human peripheral blood sample is collected.

This study obtained blood samples from 45 patients, 10 of which were clinically diagnosed as COVID-19 at the United Hospital (Wuhan, Hubei Province). The symptoms include fever, cough and obvious changes in the lungs can be seen through CT. There is no obvious respiratory failure. At the Nanyang People's Hospital (Nanyang City, Henan Province, China), 35 people were positive for the virus infection, but did not have a fever, cough or obvious changes in lung X-ray examination. According to the “Guidelines for the Diagnosis and Treatment of Novel Coronavirus (2019-nCoV) by the National Health Commission of China”, all COVID-19 patients are treated with symptomatic treatment, such as rest, antibacterial and oxygen inhalation. As a vMIP-II treatment group, 5 patients from the United Hospital were randomly selected, and vMIP-II was given intravenously through the vMIP-II treatment group: Lyophilized, each of powered vMIP-II at 250,000 active units/penicillin bottle (approximately 250 μg), dissolved in normal saline injected twice a day for 7 days was used as a course of treatment. The other 5 patients treated by the United Hospital were divided into symptomatic groups. The last 35 patients from Nanyang People’s Hospital were divided into asymptomatic groups.

Blood samples from recovering patients were taken at 7 days after patients’ blood tested negative for SARS-CoV-2. The samples were subjected to PBMC isolation, CD8^+^ T cell subgroup sorting, and co-incubation experiments involving SARS-CoV-2 S protein and vMIP-II as described below.

**1.4 Separation of PBMC**

Preparation of PBMCs: blood samples were mixed with 4 mL EDTA to prevent coagulation and centrifuged at 1500 rpm (centrifugation radius 10 cm) for 10 min. The upper plasma was extracted, and 3 mL of lymphocyte separation solution was added. The resulting mixture was centrifuged at 2500 rpm (centrifugation radius: 10 cm) for 20 min, and the supernatant was discarded. One milliliter of 1640 medium was added to resuspend 1 × 10^7^ cells/mL. Isolated normal human PBMCs were divided into five groups: a blank control group, S protein group (100 μg of S1 + S2 protein at the ratio of 2:1), and three vMIP-II treatment groups. The vMIP-II treatment group included group I (25 ng vMIP-II + S protein), group II (50 ng vMIP-II + S protein), and group III (100 ng vMIP-II + S protein). PBMCs were co-incubated with S protein and vMIP-II according to the above group doses. Each group was cultured in vitro for 12 h before use.

**1.5 Subset analysis of CD3^+^, CD4^+^, and CD8^+^ T cells among PBMCs**

Cultured cells (1.5 mL) were placed into test tubes, and 10 μL of each of four monoclonal fluorescent antibodies, including anti-human CD3-PE, CD4-FITC, CD8-APC, CD45RO-PE-Cy5, and CD62L-APC-H7 in T cells after sorting of anti-human CD3-PE (BD Biosciences, Franklin Lakes, NJ, USA) were added, and then mixed with an oscillator and stored in darkness for 15-30 min at room temperature (20-25 °C). Afterwards, 2 mL of hemolysin was added to dissolve red blood cells, and the cultures were mixed on an oscillator, stored at room temperature for 10 min in the dark, and then centrifuged at 1000 rpm for 10 min. The supernatant was discarded. Cells were washed with 1 mL of PBS buffer containing 0.1% NaN_3_ and centrifuged at 1000 rpm for 10 min. The supernatant was decanted. Fixative (300 μL) was added to resuspend the cells, which were then detected using a BD-FACsCalibur flow cytometer (BD Biosciences).

**1.6 Flow cytometric sorting of CD8^+^ T cell subtypes**

Cultured cells (1.5 mL) were placed into two test tubes, and 10 μL of each of four monoclonal fluorescent antibodies, including anti-human CD3-PE, CD4-FITC, CD8-APC, CD45RO-PE-Cy5, and CD62L-APC-H7 in T cells after sorting of anti-human CD3-PE (BD Biosciences), was added into the first test tube, which was treated according to the method in 1.3.2 and sorted on the flow cytometer. CD45RA and CD62L were used to distinguish different subgroups of CD8^+^ T cells: CD45RA^+^CD62L^+^ is the phenotype of naive CD8^+^ T cells (T_n_ cells), CD45RA^+^CD62L^-^ is the phenotype of effector CD8^+^ T cells (T_E_ cells), CD45RA^-^CD62L^+^ is the phenotype of central memory CD8^+^ T cells (T_CM_ cells); and CD45RA^-^CD62L^-^ is the phenotype of effector memory CD8^+^ T cells (T_EM_ cells). In the second test tube, 10 μL of each of three kinds of monoclonal fluorescent antibodies, including CD8-APC, PD-1-PE, and Tim-3-APC-H7 (BD Biosciences), were applied in the same manner as above and sorted on the flow cytometer. The highly expressed inhibitory molecules PD-1 and Tim-3 were used to distinguish exhausted CD8^+^ T cells (T_EX_ cells).

**1.7 Cytokine detection of specific CD8^+^ T cells induced by S protein**

Cultured cells (1.5 mL) were placed into a test tube, and PMA (50 mg/L), ionomycin (750 mg/L), and blocker BFA (1×) were added. Then the cells were incubated at 37 °C under 5% CO_2_ for 6 h. Each group of cells was equally divided into five 1.5 mL centrifuge tubes and centrifuged at 1500 rpm for 5 min. The supernatant was discarded and the cells were washed with PBS. The flow cytometry surface antibody CD8-APC-Cy7 was added to the cells in the dark after the supernatant was discarded. The cells were incubated at 4 °C for 20 min in the dark. The cells were then fixed with 200 μL of 4% paraformaldehyde. After fixing at 4 °C in the dark for 30 min, each tube was centrifuged at 500 rpm for 5 min, and the supernatant was discarded. After 200 μL film-breaking agent was added, the cells were incubated at 4 ℃ in the dark for 1 h, and then centrifuged at 1500 rpm for 5 min. The supernatant was discarded. Five tubes from each group were protected from light, and intracellular antibodies, including IFN-γ-APC, TNF-α-APC, IL2-APC, IL4-APC, and granzyme-APC (BD Biosciences) were added to the tubes and incubated at 4 ℃ for 20 min. Flow cytometry was used to detect cytokine secretion.

**1.8 RNA-seq**

Transcriptome sequencing was performed by Shanghai Kangcheng Biotechnology Co., Ltd. (Shanghai, China) The sequencing platform used was Illumina Hiseq 2500 V4, the sequencing mode was 125PE, and the samples were RNA-seq libraries of CD8^+^ T cell populations from the S protein group and 100 ng/mL vMIP-II+ S protein group. Construction of RNA-seq library: total RNA was enriched with Oligo (dT). The RNA was randomly cut into 200 nt fragments, random primer hexamers were reverse transcribed into cDNA, end repair was performed, and adenine was added. After the adapter was added, PCR amplification enabled library construction.

Sample libraries were mixed according to Illumina standards to prepare clusters. One end of the replication chain was fixed on the chip, and the other end was randomly fixed to complement another nearby primer and form a “bridge”. The single strand of the formed bridge used the surrounding primers as amplification primers, which were amplified on the surface of the chip to become double strands, and then denatured to single strands. The next round of the amplification reaction was then performed. After several rounds, each single molecule was amplified in large quantities to a cluster.

According to the quality control standard, short sequences containing linkers, short sequences with a proportion of N > 10%, and short sequences of low quality were removed. The remaining sequences (Q30 > 85%) were used for subsequent analysis.

**1.9 Differential gene expression analysis**

Differential gene expression analysis of effector CD8^+^ T cell samples in the 100 ng/mL vMIP-Ⅱ treatment group and S protein group was performed using the DESeq software. The P values of the results were controlled and adjusted using the methods of Benjamini and Hochberg. The adjusted P value < 0.01 and the differential expression multiple > 2 (|log_2_| > 1) were selected by DESeq and labeled as differentially expressed genes.

Functional enrichment analysis of differentially expressed genes was performed using GOseq R software package and the Gene Ontology resource (http://www.geneontology.org/). KEGG is a database that contain mined molecular-level information, especially large-scale molecular datasets generated by genome sequencing and other high-throughput experimental techniques, on the advanced functions of biological systems, such as cells, organisms, and ecosystems (http://www.genome.jp/kegg/). Statistical enrichment analysis of differentially expressed genes in the KEGG pathway was performed using KOBAS software. Pathway enrichment analysis of differentially expressed genes was used to analyze whether genes were over-represented in certain pathways, and gene enrichment factors were used to analyze the degree of pathway enrichment.

**1.10 Real-time PCR**

To verify the accuracy of the RNA-seq data, we selected some differentially expressed genes and performed relative fluorescence quantitative PCR analysis on their expression levels. Sorted CD8^+^ T cells were placed in a 5% CO_2_ cell incubator and cultured at 37 °C for 24 h. CD8^+^ T cell total RNA extraction was performed according to the cell total RNA extraction kit instructions, and a SuperScript^TM^ Preamplification System for First Strand cDNA Synthesis kit was used for cDNA synthesis. All primer sequences used in the real-time PCR reaction are listed in Supplementary Table 8. The reaction was performed in a real-time PCR machine (MiniOpticon; Bio-Rad, Hercules, CA, USA) according to the manufacturer’s instructions. The total reaction volume was 25.0 μL, including 2.0 μL of total RNA, 8.5 μL of RNase-free H_2_O, 0.5 μL each of the forward and reverse primers (10 mM/L), 12.5 μL 2× One-step SYBR RT-PCR Buffer Ⅲ, 0.5 μL TaKaRa Ex Taq HS (5 U/μL), and 0.5 μL PrimeScript RT Enzyme Mix II. The PCR reaction was performed at 95 °C for 5 min, 95 °C for 30 s, 60 °C for 45 s, and 72 °C for 45 s, for a total of 33 cycles. All experiments were performed in 3 independent biological replicates. Standard relative transcription levels were estimated using the 2^-ΔΔCt^ method.

**1.11 Western blot**

CD8^+^ T cells selected from the 100 ng/mL vMIP-II treatment group and S protein group were sonicated and centrifuged for 10 min, and the supernatant was collected and the pellet discarded. The supernatant was subjected to SDS-PAGE electrophoresis. After the electrophoretic band was obtained, a strip with a molecular weight of 45 kD was cut and transferred to the membrane. The membrane was stained with 1 × Polysine red staining solution for 5 min. After being washed with water, the membrane was dried for use. After the membrane was wetted with TBS from the bottom to the top, it was transferred to a plate containing a blocking solution and shaken for 1 h at room temperature on a decolorization shaker. The primary antibody Anti-G protein alpha 12 (Abcam, Cambridge, UK) was diluted to the appropriate concentration with TBST and added to the membrane. After incubation at room temperature for 1-2 hours, the membrane was washed with TBST twice at room temperature on a shaker for 10 min and then washed again with TBS for 10 min. A goat anti-rabbit Ig G-HRP dilution was then prepared and applied to the membrane. After being incubated at room temperature for 1-2 hours, the membrane was washed with TBST at room temperature twice on a shaker for 10 min each time and blotted dry. The membrane was then combined with the film. The tablet was pressed for 1 min and developed for 30 s. The membrane was then washed with water, allowed to dry, and then scanned further. ImageJ2x was used to determine the gray value of the internal reference gene and the target gene.

**1.12 Treatment with antisense RNA for G protein α subunit**

The S protein group was treated with antisense RNA for G protein α subunit, and then with vMIP-Ⅱ (100 ng/mL). CD8^+^ T cell subsets were detected by flow cytometry. The antisense oligonucleotide sequence of G_i_ α mRNA is 5’- ATG GTC AGC CCA GAG CCT CCG GAT GAC GCC CGA- 3’, a phosphorothioate oligonucleotide synthesized by Takara Bio (Kusatsu, Japan). CD8^+^ T cells were divided into four groups: the G_i_α At-RNA group, S protein, S protein + G_i_α At-RNA group, and S protein + G_i_α At-RNA + vMIP-Ⅱ group. CD8^+^ T cells were added to 96-well cell culture plates at 100 μL/well and cultured in an incubator at 37 ℃ under 5% CO_2_. G_i_ α ODN was added according to grouping 1 h after adding S protein, and vMIP-II 1 h after adding G_i_ α ODN. Cells were collected after 8 h for flow cytometric sorting of CD8^+^ T cell subtypes.

**1.13 Detection of intracellular calcium ion concentration**

Measurement of vMIP-II-induced rapid calcium influx and vMIP-II-induced calcium release from the intracellular calcium ion pool were performed separately. The former experiment was divided into 3 groups, with vMIP-II concentrations of 25 ng/mL, 50 ng/mL, and 100 ng/mL; the latter experiment was divided into 2 groups, with EDTA-containing and EDTA-free background solutions. All experiments were performed in triplicate holes. Cells were loaded with Fluo-3/AM, and 2 mL of cell suspension was added to each cuvette. Cuvettes were equilibrated at 37 °C for 5 min, and the excited with 488 nm UV light. The emitted light was detected at 525 nm. After reached baseline within 1-2 minutes, vMIP-II was added to the cells. The detection parameters of the fluorescence spectrophotometer were set, and the data interval was 0.1 min or 0.1 s. After 10 min of detection, data collection was stopped and a curve was drawn.

**1.14 Measurement of mitochondrial membrane potential**

The measurement of mitochondrial membrane potential was performed with a JC-1kit (Shanghai Biyuntian Co, Shanghai, China) according to the manufacturer’s instructions. After the culture medium was removed from the cells, the cells were washed once with PBS. Afterwards, 1 mL of cell culture solution and 0.5 mL of JC-1 staining working solution were added to the cells. After being shaken well, the cells were incubated at 37 °C for 20 min. Afterwards, the supernatant was removed, then the cells were washed twice with diluted JC-1 staining buffer (1×). Cell culture solution (2 mL) was added to the washed cells, and the cells were observed under a fluorescence microscope.

**1.15 Detection of cellular phosphorylated protein levels**

The effector CD8^+^ T cells sorted from the PBMCs were starved in 0.1% bovine serum RPMI 1640 medium for 1 h and stimulated with or without CCL21 (200 ng/mL). The cells were then collected and washed 3 times with cold PBS. Total protein was extracted after the cells were lysed, quantified by the Bradford method, and denatured by heating. SDS-PAGE was performed on the equal amounts of protein. The proteins were transferred to a PVDF membrane and blocked overnight with 5% skim milk powder formulated with TBS overnight. Rabbit anti-phosphorylated Akt (1:1000), Akt (1:1000), and PI3K (1:1000) primary antibodies were added to the membrane and incubated at room temperature for 2 h. The membrane was then washed with TTBS (TBS with 1 part per thousand Tween-20). The HRP-sheep anti-rabbit IgG secondary antibody (1:2000) was incubated for 1 h at room temperature. After being washing with TBST solution, the membrane was treated with luminescent solution for 3 min, blotted dry, and analyzed after developing. The gray value of the internal reference gene and the target genes were determined using ImageJ2x.

**1.16 ROS detection**

For CD8^+^ T cells selected from the 100 ng/mL vMIP-II treatment group and the S protein group, each sample was washed with PBS, and then a PBS solution containing 5μmol/L DCFH-DA was added, mixed well, and incubated at 37 °C for 30 minutes. Wash twice with PBS buffer to remove probes that have not entered the cells, and incubate at 37 °C for 30 min. Centrifuge at 8000 rpm for 2 min, and resuspend the cells in 1 ml PBS buffer. Detect the fluorescence intensity of each tube with a flow cytometer (excitation wavelength is 488 nm, emission wavelength is 530 nm), count 10,000 cells, and find the average fluorescence intensity. Observe after excitation with ultraviolet light (excitation wavelength 380 nm, absorption wavelength 420 nm) under a fluorescence microscope.

**1.17 Statistical analysis**

The statistical significance of all in vivo and in vitro studies was determined among three or more biological replicates using Prism software. A two-tailed t-test was used to determine the P value. * *P* < 0.05; ***P* < 0.01.


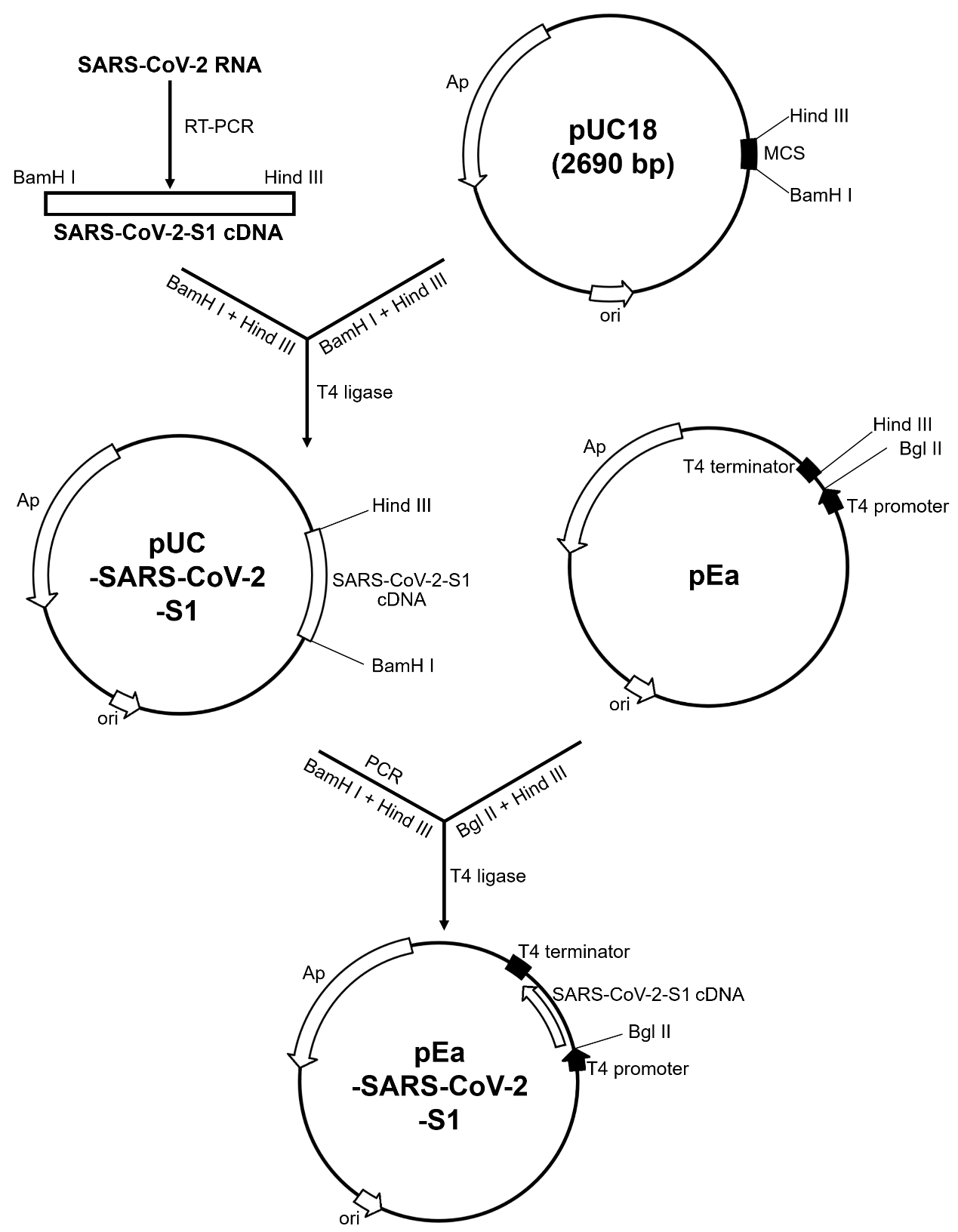


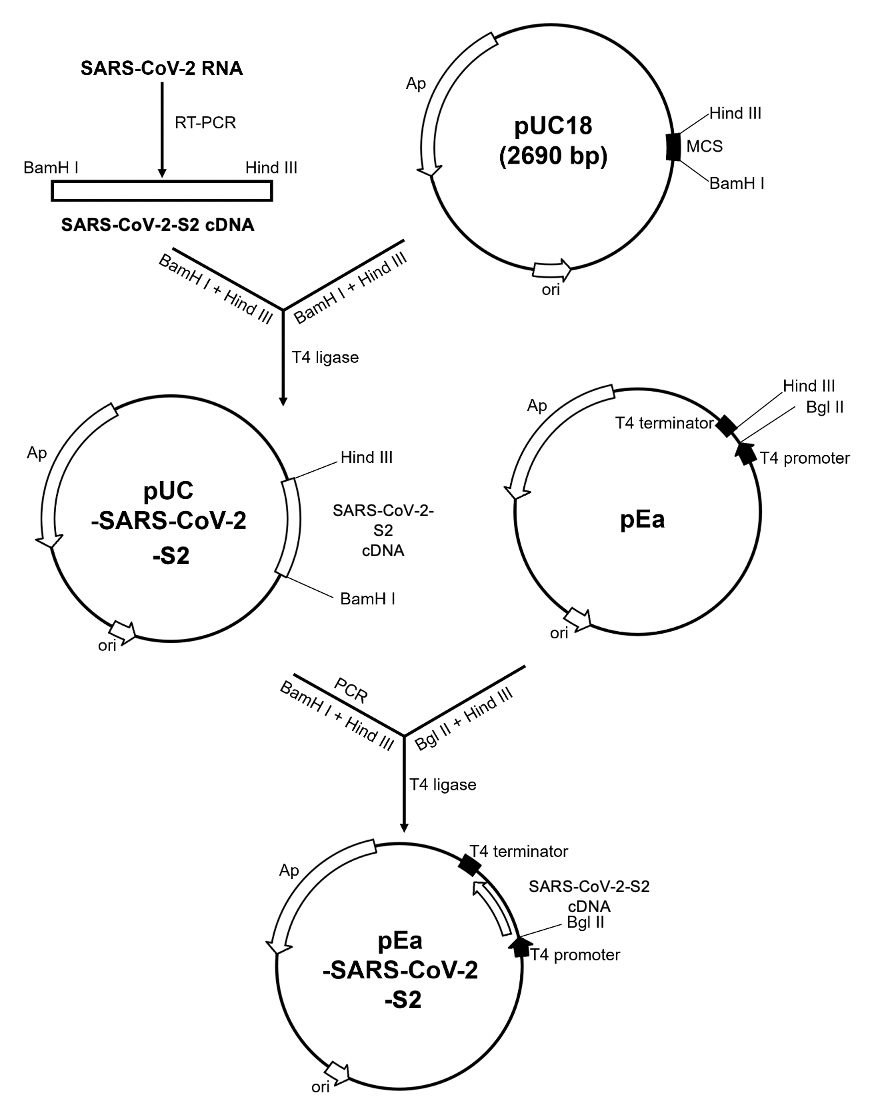


**Fig. S1 Construction scheme of pEa-SARS-CoV-2-S1 and pEa-SARS-CoV-2-S2 plasmid.**


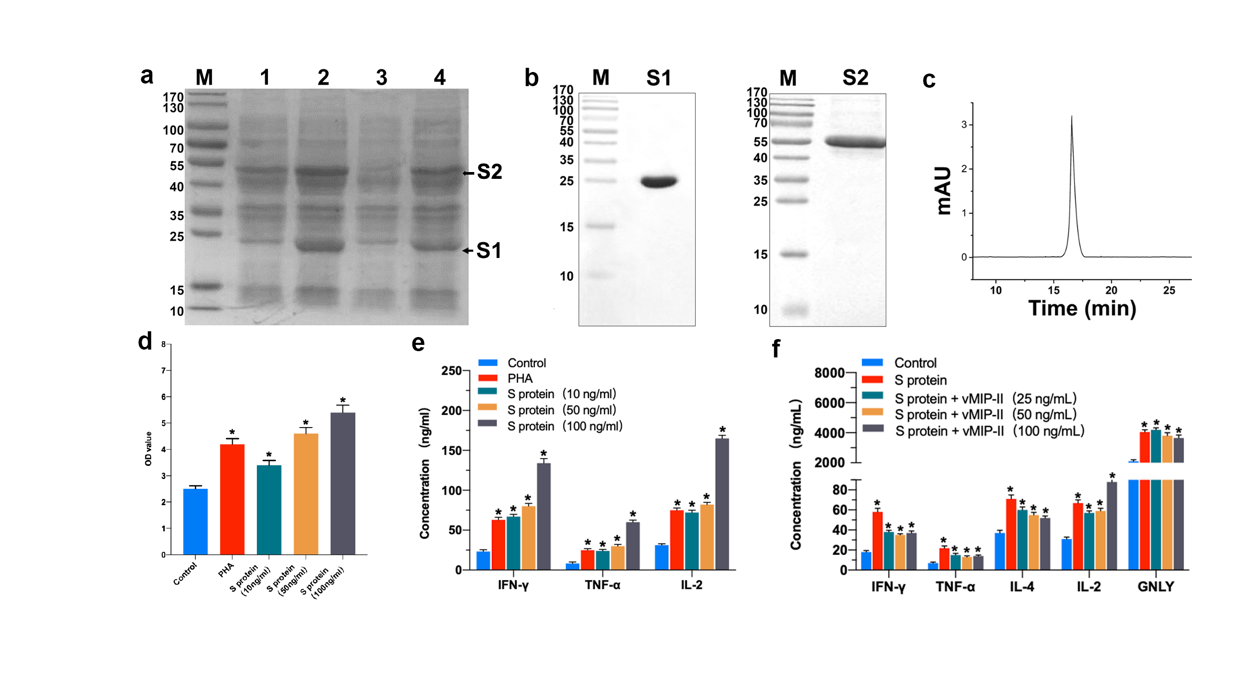


**Fig. S2 Clone, expression and antigenic activity of** **COVID-19 S protein. a**. SDS-PAGE electrophoresis of S1 cloned. S1 gene of SARS-CoV-2 were amplified and recombined into pQE-TriSystem plasmid with His tag to obtain pSARS-CoV-2-S1, which were transfected into *E.coli* M15 respectively. The *E.coli* expressing S1 protein of about 25 kDa was induced by 1mM IPTG at 37 ℃ for 4 h at 250 rpm. **b**. Electrophoretic identification of purified S1 and S2 protein. Purification > 95%. **c**. HPLC of purified S1 protein. The chromatographic conditions as follow: 20 ℃, 1 mL/min, 20 μL, wavelength: 445 nm. Mobile phase A is acetonitrile: water (v/v = 9:1) and mobile phase B is ethylacetate. **d**. Cell proliferation of PBMCs stimulated by S protein by MTT method. S protein (10, 50, 250 ng/ml) and PHA (2 μg/ml) to control, * *p*-value < 0.05. **e**. Cytokine secretion of PBMCs stimulated by different doses of S protein. There was a dose-dependent relationship. * *p*-value < 0.05; ** *p*-value < 0.01, compared with control. **f**. vMIP-II affected the cytokine secretion stimulated by S protein. Compared with the S protein group, * *p*-value < 0.05, ** *p*-value < 0.01.


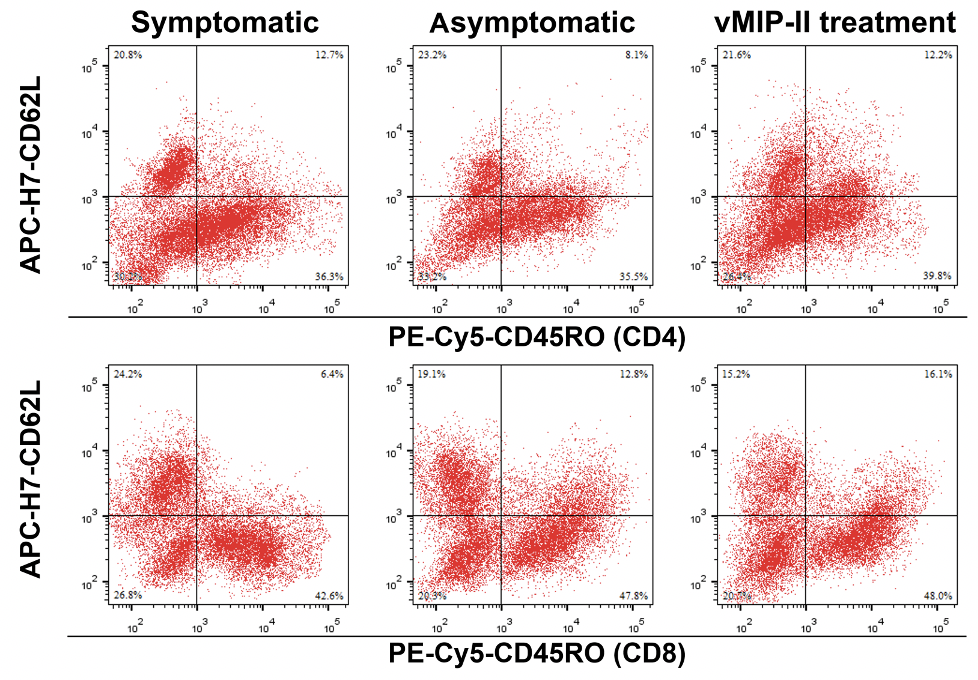


**Fig. S3 Analysis of T cell subgroups in PBMCs of convalescent patients after one week of being the virus negative.** The ratios of T_CM_ cells in the vMIP-II treatment and asymptomatic infection groups were significantly higher than that in the symptomatic infection group, although there was no significant difference in the total number of lymphocytes.


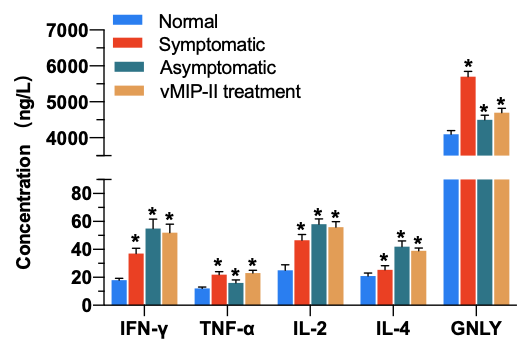


**Fig. S4 Effects of vMIP-II on the level of cytokines secretion of CD8^+^ T cells induced by S protein.** Compared with the S protein group, TNF-α and IL-4 levels were decreased in the vMIP-II co-incubation groups. * *p*-value < 0.05.

**
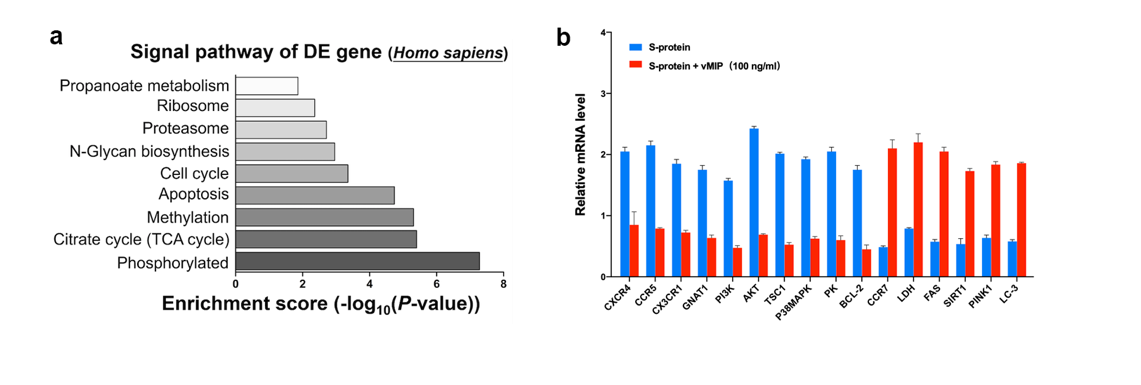
**

**Fig. S5 Analysis of KEGG pathway of differentially expressed genes and its qRT-PCR verification. a**. Analysis of KEGG pathway (Notes: The longer the bar is, the higher the enrichment degree is; or conversely). **b**. qRT-PCR verification of differentially expressed genes. Compared with S protein group, * *p* value < 0.01.

**
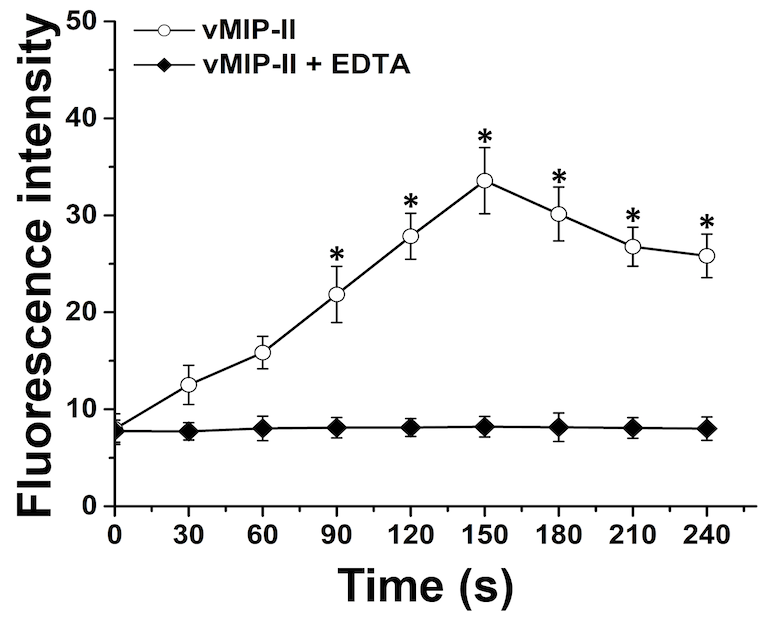
**

**Fig. S6 Measurement of vMIP-II-induced calcium release.** Compared with the control group, vMIP-II treatment can increase intracellular calcium chelation, with a * *p* value of < 0.01.


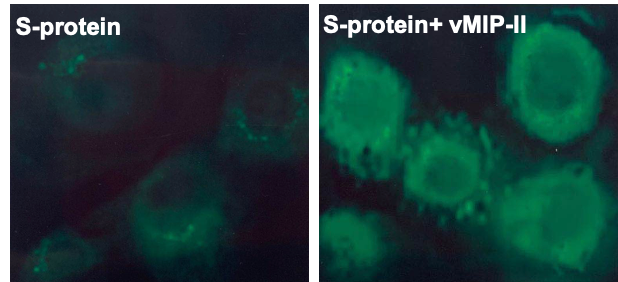


**Fig. S7 The ROS level of CD8^+^ T cells.** vMIP-II increased the ROS level of CD8^+^ T cells, the left side is the S protein control group, and the right side is the vMIP-II treatment group. bar. 20 μm, ** *p* value < 0.01.


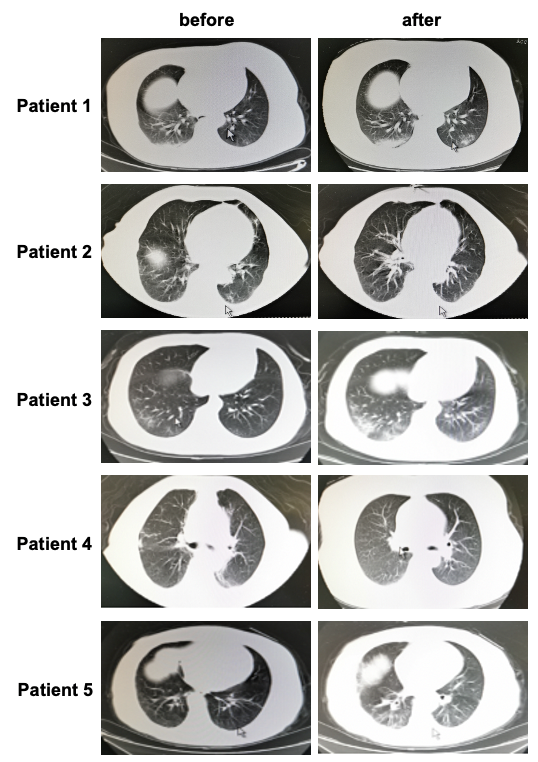


**Fig. S8 Patients lung CT.** Five common patients with SARS-CoV-2 virus were scanned by lung CT before and one week after symptomatic treatment (not with vMIP-II treatment).

**
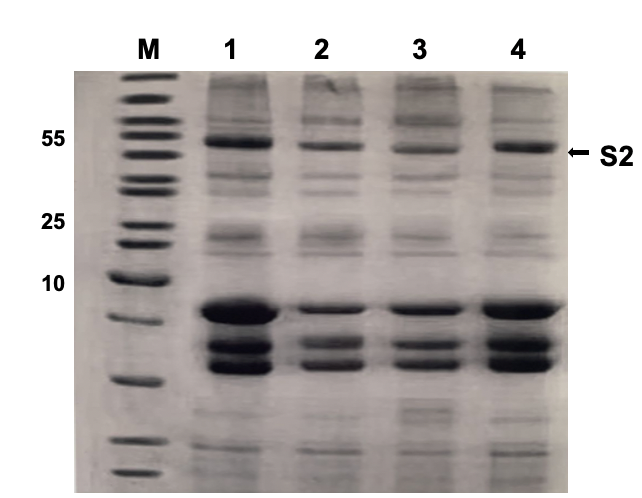
**

**Fig. S9 SDS-PAGE electrophoresis of S2 cloned.** S2 gene of SARS-CoV-2 were amplified and recombined into pQE-TriSystem plasmid with His tag to obtain and pSARS-CoV-2-S2, which were transfected into E.coli M15 respectively. The E.coli expressing S2 protein of about 55 kDa was induced by 1mM IPTGat 37 ℃ for 4 h at 250 rpm.

**Table S1. Changes in peripheral blood lymphocytes of patients after continuous**

vMIP-Ⅱ treatment for 1 week **(Mean ± SD, *n =* 5, 10^9^/L)**

| **Group** | **vMIP-II treatment** | | **Conventional treatment** | |
| --- | --- | --- | --- | --- |
|  | **Enrollment** | **After treatment** | **Enrollment** | **After treatment** |
| [Total Leucocyte Count](https://www.baidu.com/link?url=EeCtS2D-eCB0xzSR0kYQstRjY21KcajxW0SGmrOH9t0_o-8ZvVY5Zqacrf6nKWiHnZaW-zGwpxIvwXQRDzx5qI9q8njXVEyyKnDZBSg3rHTdXMuaS51YL_PfOrM--_Hs&wd=&eqid=ee2889f70014b089000000065e844543)  **（10^9^/L）** | 6.74 ± 1.93 | 7.09 ± 2.08 | 7.27 ± 1.68 | 7.68 ± 1.72 |
| [Total Lymphocyte Count](https://www.baidu.com/link?url=BobRrfj8Cb1CPNG94MFDSyunZxsXcnEf2RI1oT5-aOtWuk061st1f40yoWDT6Y4UgufcS9OXItvgYSwd7MkiFikim_Ssb5zE86K1MimNirnIGFBiaZ-mLPMOjLk_4l6F&wd=&eqid=aaf6573b0014b36b000000065e8445cc)  **（10^9^/L）** | 0.90 ± 1.21 | 1.51 ± 1.17* | 0.83 ± 1.15 | 1.28 ± 2.23 |
| [D dimer](https://www.baidu.com/link?url=IK3TwxOnBJFKTgXZW2y0T0Z0zp7f4_jQ6aflFEdXD_T-uNxSHNXW76uc2DclZertKm8kor1UAoT4JfwfoQGqerMKgAoJLHMQ7VkW_AHByQ7&wd=&eqid=9b744e9c00030311000000065e844614)  **（mg/L）** | 1.94 ± 0.17 | 0.85 ± 0.07* | 2.08 ± 0.21 | 0.99 ± 0.09 |
| White blood cells**（10^9^/L）** | 3.23 ± 0.13 | 4.79 ± 0.8* | 3.78 ± 0.17 | 3.86 ± 0.23 |
| Eosinophils  **（10^9^/L）** | 0.01 ± 0.01 | 0.07 ± 0.01* | 0.01 ± 0.01 | 0.01 ± 0.01 |
| neutrophil granulocyte  **(%)** | 73 ± 3 | 66 ± 1.2* | 70.2 ± 2 | 70.3 ± 1.4 |
| Red Cell volume  Distribution Width  **(%)** | 37.8 ± 2.1 | 40.2 ± 1.1* | 37.3 ± 2.3 | 36.7 ± 1.3 |

Note: * indicates *P* < *0.05* compared with the conventional treatment group;

** indicates *P* < *0.01* compared with the conventional treatment group.

**Table S2. Analysis of negative conversion time of patients**

|  | **vMIP-II treatment** | | | | | **Conventional treatment** | | | | |
| --- | --- | --- | --- | --- | --- | --- | --- | --- | --- | --- |
| **Patient** | 1 | 2 | 3 | 4 | 5 | 1 | 2 | 3 | 4 | 5 |
| **Virus to be negative time (days)** | 14 | 15 | 16 | 16 | 18 | 20 | 21 | 25 | 22 | 22 |
| **Median time (days)** | 16 | | | | | 22 | | | | |
| **SD** | 2.2 | | | | | 3.5 | | | | |

**Table S3. Analysis of T subpopulations in PBMCs of recovery patients**

| **project** | **Diseased group（%）** | **non-symptoms group（%）** | **vMIP-II treatment group（%）** |
| --- | --- | --- | --- |
| Lymphocyte | 30.9 ± 3.86 | 34.2 ± 4.59 | 35.8 ± 5.14 |
| CD3^+^/Lymphocyte | 60.1 ± 6.22 | 70.2 ± 7.28* | 69.4 ± 6.68* |
| CD3^+^CD4^+^ | 33.3 ± 6.12 | 39.2 ± 7.62* | 38.5 ± 6.27* |
| CD3^+^CD8^+^ | 25.4 ± 7.55 | 27.4 ± 8.41 | 26.5 ± 7.83 |
| CD45RA^+^/CD4 | 47.3 ± 10.2 | 55.9 ± 9.62* | 50.6 ± 9.13 |
| CD45RO^+^/CD4 | 50.2 ± 9.27 | 44.3 ± 8.8 | 48.3 ± 9.61 |
| CD4^+^ T_CM_ | 13.1 ± 2.41 | 8.7 ± 3.15 | 11.9 ± 2.21 |
| CD4^+^ T_EM_ | 38.4 ± 3.15 | 36.2 ± 3.22 | 37.1 ± 5.66 |
| CD45RA^+^/CD8 | 49.1 ± 7.19 | 37.2 ± 9.17* | 35.2 ± 7.66* |
| CD45RO^+^/CD8 | 52.8 ± 8.33 | 61.8 ± 8.42* | 63.8 ± 9.34* |
| CD8^+^ T_CM_ | 7.12 ± 2.12 | 14.3 ± 5.12** | 17.1 ± 4.12** |
| CD8^+^ T_EM_ | 41.9 ± 5.22 | 48.6 ± 6.31* | 47.6 ± 7.22* |

Note: Compared with the common symptomatic group, ** P < 0.05*; *** P < 0.01*

**Table S4. Changes in the subsets of memory CD8^+^ T cells (%. Mean ± SD. n = 3)**

| Treatment | Memory CD8^+^ T | Percentage of CD8^+^Tcm | Percentage of CD8^+^ Tem | Percentage of CD8^+^ T_EX_ |
| --- | --- | --- | --- | --- |
| Untreated control | 73.99 ± 4.56 | 9.33 ± 4.54 | 86.20 ± 5.18 | 4.05 ± 1.17 |
| 25ng/ml | 73.92 ±3.33 | 17.22 ± 2.75* | 81.53 ± 6.17 | 6.83 ± 1.12* |
| 50ng/ml | 71.01 ± 4.63 | 23.85 ± 4.12** | 75.17 ± 6.44 | 5.18 ± 1.18* |
| 100 ng/ml | 70.18 ± 3.45 | 29.39 ± 2.44** | 71.19 ± 5.45 | 3.85 ± 1.69** |
| Sprotein  (100 ng/ml) | 66.30 ± 2.56 | 8.19 ± 1.48 | 89.33 ± 7.62 | 12.94 ± 1.88* |

Note: Compared with the control group, **P < 0.05,* ***P < 0.01*

**Table S5. Gene ontology analysis for significantly altered genes-biological process**

| Biological Progress | Count | P-value |
| --- | --- | --- |
| Phosphorylation process | 20 | 3.3E-06 |
| Cell apoptosis | 15 | 4.8E-04 |
| Cell differentiation | 11 | 6.1E-04 |
| Glucose metabolic process | 9 | 1.2E-03 |
| Cell localization and metabolism | 9 | 3.4E-03 |
| [Regulation of transcription, DNA-templated](http://www.ebi.ac.uk/QuickGO/GTerm?id=GO:0006355) | 9 | 5.3E-03 |
| [Regulation of mitophagy](http://www.ebi.ac.uk/QuickGO/GTerm?id=GO:1903146) | 7 | 4.1E-03 |
| [Cellular response to mechanical stimulus](http://www.ebi.ac.uk/QuickGO/GTerm?id=GO:0071260) | 6 | 2.8E-02 |
| [Systerm](http://www.ebi.ac.uk/QuickGO/GTerm?id=GO:0042593) process | 6 | 3.2E-02 |
| [Transcription from RNA polymerase II promoter](http://www.ebi.ac.uk/QuickGO/GTerm?id=GO:0045944) | 5 | 3.8E-02 |

**Table S6. Gene ontology analysis for significantly altered genes-cellular component**

| Cellular Component | Count | P-value |
| --- | --- | --- |
| Cytoplasm | 26 | 5.3E-03 |
| Intracellular membrane-bounded organelle | 16 | 2.8E-04 |
| Endoplasmic reticulum | 12 | 4.5E-04 |
| Mitochondrion | 12 | 6.2E-04 |
| Extracellular exosome | 11 | 3.1E-03 |
| Nucleoplasm | 10 | 3.3E-03 |
| ER to Golgi transport vesicle membrane | 6 | 1.2E-02 |
| RNA polymerase II transcription factor complex | 6 | 3.1E-02 |
| Receptor complex | 5 | 2.8E-02 |
| Peroxisome | 4 | 6.1E-02 |

**Table S7. Gene ontology analysis for significantly altered genes-molecular function**

| Molecular function | Count | P-value |
| --- | --- | --- |
| Protein binding | 28 | 8.5E-06 |
| Sequence-specific DNA binding | 11 | 2.0E-05 |
| Transcription factor activity | 10 | 1.3E-05 |
| DNA binding | 10 | 3.1E-04 |
| Identical protein binding | 8 | 2.8E-04 |
| Transcription regulatory region DNA binding | 7 | 4.6E-04 |
| Cytokine activity | 6 | 4.9E-03 |
| Receptor binding | 6 | 1.5E-02 |
| Oxidoreductase activity | 6 | 3.6E-02 |
| Steroid hormone receptor activity | 5 | 1.7E-02 |
| RNA polymerase II transcription factor activity | 5 | 7.9E-02 |

**Table S8. Primer sequences for quantitative PCR reactions**

| Primer name | Primer sequence (5’- 3’) |
| --- | --- |
| CXCR4 Forward | CTCCTCTTTGTCATCACGCTTCC |
| CXCR4 Reverse | GGATGAGGACACTGCTGTAGAG |
| CX3CR1Forward | CACAAAGGAGCAGGCATGGAAG |
| CX3CR1 Reverse | CAGGTTCTCTGTAGACACAAGGC |
| CCR5 Forward | TCTCTTCTGGGCTCCCTACAAC |
| CCR5 Reverse | CCAAGAGTCTCTGTCACCTGCA |
| CCR7 Forward | CAACATCACCAGTAGCACCTGTG |
| CCR7 Reverse | TGCGGAACTTGACGCCGATGAA |
| GNAI1 Forward | CCTGTTTCAACAGATCCCGAGAG |
| GNAI1 Reverse | TCGGGATGTAATTTGGTTGAGCTA |
| PI3K Forward | GAGAGCTTGGAGGACGATGATG |
| PI3K Reverse | CCACGCTTCAGCAGAAATCTGG |
| P38MAPK Forward | GAACAAGACCGTCTGGGAGGTGC |
| P38MAPK Reverse | TTGGCGTGAATGATGGACTGAAA |
| PINK1 Forward | ATCAGTAGCATCTAGCATAC |
| PINK1 Reverse | GATCACTGATCAGATCTATCC |
| LC3 Forward | AGCTCTGAAGGCAACAGCAACA |
| LC3 Reverse | GCTCCATGCAGGTAGCAGGAA |
| SIRT1 Reverse | CTGTTTCCTGTGGGATACCTGACT |
| SIRT1 Reverse | ATCGAACATGGCTTGAGGATCT |
| TSC1 Forward | GTCACGACCGTAGGAGAAGC |
| TSC1 Reverse | GAATCAACCCCACAGAGCAT |
| AKT Forward | TGGACTACCTGCACTCGGAGAA |
| AKT Reverse | GTGCCGCAAAAGGTCTTCATGG |
| FAS Forward | GGACCCAGAATACCAAGTGCAG |
| FAS Reverse | GTTGCTGGTGAGTGTGCATTCC |
| Bcl-2 Forward | ACGACCTCAACGCACAGTACGA |
| Bcl-2 Reverse | CCTAATTGGGCTCCATCTCGGG |
| PK Forward | TCAGGATGCGTCCACCAAGAAG |
| PK Reverse | TGTGTCCACGGCGGCAATCATC |
| LDH Forward | ATGAAAAAAATTATTGTTATAGGTATGGGA |
| LDH Reverse | TTATTCAAAATCCATATCAATGTTTATTAATT |
| GAPDH Forward | GTCTCCTCTGACTTCAACAGCG |
| GAPDH Reverse | ACCACCCTGTTGCTGTAGCCAA |
