# Supplementary material for: Study on the promotion of lymphocytes in patients with COVID-19 by broad-spectrum chemokine receptor inhibitor vMIP-II and its Mechanism of signal transmission in vitro
Source: Signal Transduct Target Ther. 2021 Mar 2;6:104. doi: 10.1038/s41392-021-00516-4 (PMC7921284; doi:10.1038/s41392-021-00516-4)
Supplement: Supplementary file 2 — instructions for the use of vMIP freeze-dry powder injection [file 41392_2021_516_MOESM2_ESM.pdf]

Recombinant viral macrophage inflammatory protein drug Instructions

[Drug Name]

Common name: Recombinant viral macrophage inflammatory protein for injection

Product name: Hai mi pu (English trade name: vMIP)

English abbreviation: vMIP

Chinese Pinyin: zhu she yong chong zu bing du ju shi xi bao yang xing dan bai

Main components: viral macrophage inflammatory protein 250,000 units / branch (or 0.25mg)

Ingredients: 1% human serum albumin (10g / 100 sticks)

[Character]

White lyophilized powder

[Pharmacology and Toxicology]

Pharmacology

Mechanism of action

Viral macrophage inflammatory protein (vMIP) is a homologue of chemokine derived from the recombinant natural human herpesvirus 8 gene. Chemokine receptors are the pathogenesis of disease occurrence and development. It has been proved that vMIP can effectively inhibit the process of HIV infecting target cells through chemokine receptors. VMIP, as a receptor blocking factor, blocks the binding of HIV (HIV) to target cells CCR5 and CXCR4, which is antagonistic to HIV infection The target cells are more ideal natural virus-derived co-receptor inhibitors, which can prevent and treat HIV infection and AIDS. vMIP obviously promotes virus-specific immunity, and can also be used to prevent and control other viral infections.

Efficacy of characteristics

In vitro experiments using HIV-sensitive CD4 + T lymphocyte-MT4 cells vMIP can achieve a cell protection rate of 90%. After vMIP, the virus replication level in human peripheral blood mononuclear cells is significantly reduced, and cynomolgus monkeys are infected with SIVmac251 animal model drugs Efficacy studies showed changes in viral load. Virus detection results in different vMIP dose groups showed that the viral load level in plasma decreased faster than the control group, showing a significant volume-effect relationship. In different doses of peripheral blood mononuclear cells Compared with the AZT + 3TC group, the viral load level decreased rapidly, and the virus copy number was significantly lower than that of the treatment control group at 70 days and 84 days, indicating that vMIP has the effect of antagonizing monkey HIV (SIV) replication. There was no significant change in the total number of leukocytes in the blood routine examination of the vMIP group, and

the proportion of lymphocytes in the leukocyte classification increased.The total number of CD4 + cells and the ratio of CD4 + / CD8 + in the vMIP group rose to normal water half 56 days after treatment. Obvious protection.

Post-exposure prophylaxis (PEP)

This product has a significant blocking effect on the entry of HIV virus into target cells. It is recommended to use this product to prevent HIV (HIV) in the blood event of accidental exposure to HIV infection, such as needle stick injury. The duration of medication should last 4 weeks.

Preclinical safety

Acute toxicity

The maximum tolerable dose of intravenous injection in mice MTD> 40mg / Kg, cynomolgus monkey intravenous injection MTD> 12.5mg / Kg. Both are 2500 times the effective dose, no animal toxicity was seen.

When cynomolgus monkeys were intravenously injected with 1250ug / Kg body weight (250 times the pharmacodynamic effective dose of 5mg / Kg), individual animals developed lymphoid follicular hyperplasia, and the size of the follicles varied, mainly in the cortical area, the germinal center was enlarged, and the peripheral blood was visible Component indicators check lymphocyte classification and total increase. The detection results of apoptosis in lymphoid organs showed that there was no apoptosis that caused lymphoid organs, and the results showed that vMIP caused lymphocyte proliferation in lymphoid tissues.

Mutagenic effect

In vitro microbial mutation (Ames) test, in vivo micronucleus test and mammalian cultured cell chromosome aberration test have not shown that vMIP is genotoxic and mutagenic.

Reproductive toxicity

The dosage of SD rats in the teratogenic test was 630ug / Kg / day, and no toxic reaction to fetal rats was seen.

[Pharmacokinetics]

Absorption: The plasma concentration reaches a peak 5 minutes after a single administration in SD rats and cynomolgus monkeys. In the therapeutic dose and the range greater than 100 times the therapeutic dose, the absorption and elimination process of the drug has linear kinetic characteristics.

Distribution: Non-highly bound to plasma protein (this combination is 55.6%) eliminates faster in SD rats, with an average elimination half-life of 3.0h, and the drug concentration in cynomolgus monkeys decreases slowly, with an average elimination half-life of 7.49h; In the target organs such as the lymphatic system of rats and cynomolgus monkeys, the distribution of drugs can quickly reach equilibrium. The vMIP in the bone marrow and intestinal mucosal lymph nodes also stays for a long time, and the vMIP content of other organs does not change much.

Metabolism: Excreted mainly from the urine, less excretion.

[Indications]

1. HIV-infected patients with incubation positive for clinical tests;
2. People living with HIV and patients at the onset of illness;
3. Prevention of HIV-susceptible people;
4. Other viral infectious diseases;

This product is suitable for adults and children with the above indications.

[Dosage]

The treatment of this product should be conducted under the guidance and monitoring of a physician with experience in treating HIV infection.

Adults: The safe dose range of the drug is 2 ~ 90ug / Kg body weight, the recommended dose starts at 0.5mg / Kg / day, the time is 1mg / intravenous injection after dissolving with 1.0ml of sterile water for injection; children (3 years old and above)): The dose is adjusted according to the adult dose and the child. The formula is shown below: Child dose = adult dose x child weight (Kg) / 50 (Kg), but it is easy to appear that the child finds that the weight is low and the weight of the elderly is high If the dosage of children exceeds the adult dosage based on body weight, the adult dosage is 20ug / Kg / day.

[Drug combination]

This product is an entry inhibitor for HIV infection and can be used in combination with reverse transcriptase inhibitors and protease inhibitors such as lamivudine, zidovudine and other mechanisms of action.

[Adverse reactions]

The use of this product rarely causes adverse reactions.

[Taboo]

Those with severe allergies to albumin are prohibited.

[Precautions]

1. After the drug solution is dissolved, turbidity, precipitation, undissolved foreign objects or cracks in the bottle, loose bottle cap, expired and invalid conditions cannot be used.
2. After the product is turned on, one intravenous injection should be completed without score or injection to the second person.
3. If an uncomfortable reaction is found during the injection process, the injection should be stopped immediately.
4. Freezing is strictly prohibited during transportation and storage.

[Pregnancy and lactation medication]

There has not been enough and strictly controlled research on pregnant women and lactating women. Only when the possible benefit exceeds the harm to the fetus, can it be used during pregnancy. It is not known to lactating women whether this product is excreted through milk, given Excreted from human breast milk, if

breastfeeding women are using this product, it is recommended to interrupt breastfeeding. If necessary, it should be used under the guidance and close observation of a physician.

[Child medication]

This product is suitable for children aged 3 years and over (see Usage and Dosage for Children). It has not been studied in patients under 3 years of age

[Drug interaction]

This product should not be used in combination with vasoconstrictor drugs, proteolytic drugs or alcohol-containing solvents.

[Overdose]

There is no adverse reaction caused by excessive use of this product.

[Specification] Specific activity is 25×10^5U / branch (or 0.25mg / branch)

[Validity period] 2 years

[Storage] Store at 4℃, keep it sealed to prevent moisture.

[Drug Registration Certificate Number]

[manufacturer]

Institute of Genomic Medicine, School of Pharmacy, Jinan University
Address: No. 601, Huangpu Avenue West, Tianhe District, Guangzhou
Phone: 020-38375022, Fax: 020-89856367

病毒巨噬细胞炎性蛋白(vMIP)使用说明书

[药品名称]

通用名: 注射用重组病毒巨噬细胞炎性蛋自

商品名: 海米普 (英文商品名 vMIP)

英文名: recombinant viral macrophage inflammatory protein for injection

英文缩写: vMIP

汉语拼音: zhu she yong chong zu bing du ju shi xi bao yang xing dan bai

主要组成成分: 病毒巨噬细胞炎性蛋白 25 万单位/支(或 0.25mg）

辅料: 1%人血清白蛋白(10g/100 支)

[性状]

白色冻干粉末状

[药理毒理]

药理学

作用机理

病毒巨噬细胞炎性蛋白(vMIP)是重组的天然病毒人疱疹病毒 8 基因来源的趋化因子同源物,它可以封闭和抑制位于细胞表面的趋化因了受体,可阻断以利用趋化因子受体为发病机理的疾病的发生和发展。现已证明, vMIP 可有效地抑制 HIV 通过趋化因子受体感染靶细胞过程, vMIP 作为受体封闭因子,阻断了艾滋病病毒(HIV)与靶细胞 CCR5 和 CXCR4 的结合作用,是拮抗 HIV 感染靶细胞更理想的天然病毒来源的共受体抑制因子,对 HIV 感染和艾滋病者具有防治作用。 vMIP 对病毒特异性免疫有明显促进作用，也可用于其他病毒感染的防治。

药效特点

体外实验用 HIV 敏感的 CD4+T 淋巴细胞-MT4 细胞 vMIP 的细胞保护率可达到 90%, vMIP 作用后人外周血单个核细胞内的病毒复制水平明显降低,食蟹猴感染 SIVmac251 的动物模型药效学研究可见病毒载量的变化，不同 vMIP 剂量组的病毒检测结果显示血浆中病毒载量水平相较于对照组降低较快, 呈明显量-效应关系,不同剂量的外周血单个核细胞内病毒载量水平与 AZT+3TC 组比较，降低均较快,至 70 天和 84 天时病毒拷贝数明显低于治疗对照组,说明 vMIP 具有拮抗猴艾滋病病毒(SIV)复制的作用。对 vMIP 组血常规检查动物白细胞总数无

明显变化,白细胞分类中淋巴细胞比例增高, vMIP 组的CD4+细胞总数和CD4+/CD8+比值用药后56天回升至正常水半, vMIP 对其作用的靶细胞有明显保护作用。

暴露后预防(PEP)

本品对 HIV 病毒进入靶细胞有明显阻断作用,推荐在意外接触HIV 感染的血液事件中如针刺伤等建议用本品对艾滋病病毒(HIV)进行预防,用药时间应持续4周。

临床前安全性

急性毒性

小鼠静脉注射的最大耐受量MTD>40mg/Kg,食蟹猴静脉注射的 MTD>12.5mg/Kg.均为有效剂量的2500倍,未见动物出现毒性反应。

食蟹猴静脉注射1250ug/Kg体重(为药效学有效剂量5mg/Kg的250倍)时个别动物出现淋巴滤泡增生,滤泡大小不等, 主要位于皮质区,生发中心扩大, 外周血有形成份指标检查淋巴细胞分类和总数增加。淋巴器官的细胞凋亡检测结果显示，未见引起淋巴器官的细胞凋亡,结果表明vMIP 引起淋巴组织的淋巴细胞增生。

致突变作用

在体外微生物突变(Ames)试验,体内微核试验和哺乳动物培养细胞染色体畸变试验等研究中均无显示vMIP 有遗传毒性和致突变性。

生殖毒性

SD大鼠的致畸胎试验时用药剂量为630ug/Kg/天,未见对胎鼠的有毒性反应。

[药代动力学]

吸收: 在SD 大鼠体内和食蟹猴体内单次给药后5min 血药浓度达高峰,在治疗剂量及大于治疗剂量100倍的范围，药物的吸收和消除过程呈线性动力学特征。

分布: 与血浆蛋白的非高度结合(本结合55.6%)在SD大鼠体内消除较快,平均消除半衰期为3.0h，在食蟹猴体内药物浓度下降缓慢,平均消除半衰期为7.49h; SD大鼠、食蟹猴的淋巴系统等靶器官中, 药物的分布可很快达到平衡,在骨髓和肠粘膜淋巴结中的vMIP 也滞留较长时间，其他脏器的 vMIP 含量变化不大。

代谢: 主要从尿中排泄,粪排泄较少.

[适应症]

1. HIV感染者临床检测阳性的潜伏期患者；

2. HIV感染者和发病期患者；

3. HIV 易感人群的预防；

4. 其他病毒感染性疾病；

本品适用于以上适应症的成人及儿童。

[用法用量]

本品的治疗应在有治疗 HIV 感染经验的内科医生指导和监测下进行。

成人: 药物的安全剂量范围为 2~90ug/Kg 体重,推荐剂量以 0.5mg/Kg/天开始,用时 1mg/支用 1.0ml 无菌注射用水溶解后静脉注射；儿童(3 岁及 3 岁以上):剂量根据成人剂量和儿童作相应的调整，公式见下:儿童剂量=成人剂量 x 儿童体重(Kg)/50(Kg)，但易出现幼儿求得体重偏低,年长儿体重偏高的现象,若按体重计算儿童剂量超过成人剂量时,则以成人剂量 20ug/Kg/天为限。

[联合用药]

本品为 HIV 感染的进入抑制剂,可与其他作用机制的逆转录酶抑制剂、蛋白酶抑制剂如拉米夫定、齐多夫定等联合应用。

[不良反应]

使用本品极少产生不良反应。

[禁忌]

对白蛋白有严重过敏者禁用。

[注意事项]

1.药液溶解后出现混浊、沉淀、不溶解的异物或瓶子有裂纹、瓶盖松动、过期失效等情况不可使用。

2.本品开启后应一次静注完毕,不得分次或给第二人注射。

3.注射过程中如发现病人有不适反应,应立即停止注射。

4.运输及贮存过程中严禁冻结。

[孕妇及哺乳期用药]

尚未对孕妇及哺乳期妇女进行足够的和严格对照的研究,只有在可能的受益超过对胎儿的危害时方可在妊娠期用药. 对哺乳期妇女尚不知本品是否经乳汁排泄,鉴于许多药物从人乳汁中排泄,如果哺乳期妇女正在使用本品,建议中断哺乳。如有必要应用时,应在医师指导和严密观察下使用。

[儿童用药]

本品适用于 3 岁及 3 岁以上的儿童患者(见儿童患者用法及用量)，尚未在 3 岁以下患者中进行研究

[药物的相互作用]

本品不宜与血管收缩药、蛋白酶水解药或含有酒精溶剂的混合液使用。

[药物过量]

本品未见有过量使用产生不良反应。

[规格] 比活性为 25X10^{5}U/支 （或 0.25mg/支）

[有效期] 2 年

[贮存] 4℃保存,保持密封,防止潮湿.

[药品注册证号]

[生产企业]

暨南大学药学院基因组药物研究所

地址:广州 市天河区黄埔大道西 601 号

电话: 020-38375022,传真:020-89856367
