# Supplementary material for: Study on the promotion of lymphocytes in patients with COVID-19 by broad-spectrum chemokine receptor inhibitor vMIP-II and its Mechanism of signal transmission in vitro
Source: Signal Transduct Target Ther. 2021 Mar 2;6:104. doi: 10.1038/s41392-021-00516-4 (PMC7921284; doi:10.1038/s41392-021-00516-4)
Supplement: Supplementary file 3 — Summary of clinical trial vMIP to COVID-9 in Union Hospital [file 41392_2021_516_MOESM3_ESM.pdf]

<u>**Medical Ethics Committee of Union Hospital affiliated to Tongji Medical College of Huazhong University of Science and Technology**</u>

Approvals of Medical Ethics Committee of Union Hospital affiliated to Tongji Medical College of Huazhong University of Science and Technology

[2020] Ethical examination number(0006) Date of examination : 2020.02.06

Protocol Title/Number	Efficacy and safety of recombinant virus macrophage inflammatory protein (vMIP) for aerosol inhalation injection in the treatment of novel coronavirus pneumonia: a single-arm clinical trial
Sponsor/Study product	Guangzhou Yuanyuan Biotechnology Co., Ltd.; Institute of genetic Medicine Group, Jinan University
Approval Number by NMPA	NA
PI/site	Hu Bo, Li Wei/Neurology Department, Infection department
List of reviewed documents with Version No.<br><br>Please refer to the attached list for details.	
The way of ethical examination	☐ Routine meeting ☑ Emergency meeting

The Ethics Committee received the application for the preliminary examination of the project submitted by the main researchers Hu Bo and Li Wei. After examination, the Medical Ethics Committee considered that the project met the ethical requirements and agreed to the implementation of the project.

The ethical approval is valid for one year, and the progress of the research will be informed to the Ethics Committee every year after the start of the study. At the end of the study, the termination report of the study shall be submitted to the Ethics Committee.

Medical Ethics Committee of Union Hospital affiliated to Tongji Medical College of Huazhong University of Science and Technology (sealed):

Signature of director or deputy director:

Date:

Adress: No. 1277 Jiefang Avenue, Wuhan City, Hubei Province Postal code: 43000

Telephone: 027--85726685 Fax: 0086-27-85726685 E-mail: whunionlunli@126.com

华中科技大学同济医学院附属协和医院医学伦理委员会审批件

[2020]伦审字（0006）号 **审查日期:2020.02.06**

研究方案名称/编号<br>Protocol Title/Number	雾化吸入注射用重组病毒巨噬细胞炎性蛋白(vMIP)治疗新型冠状病毒感染肺炎有效性和安全性的单臂临床试验
申办者（或合作单位）/试验产品<br>Sponsor/Study product	广州溯原生物科技股份有限公司；<br>暨南大学基因药物组药物研究所
NMPA 批件号 Approval Number by NMPA	NA
主要研究者/单位 PI/site	胡波、李伟/神经内科、感染科
审查文件（含版本号）如下：List of reviewed documents with Version No.<br><br>**详见附件清单**	
伦理审查方式	☐例行会议 ☑紧急会议

伦理委员会收到主要研究者胡波、李伟教授提交的该项目初审申请，经审查医学伦理委员会认为该项目符合伦理学要求，同意该项目实施。

伦理批件有效期为1年，研究启动后每年将研究进展情况向伦理委员会通报，研究结束时，需向伦理委员会提交研究终止报告。

华中科技大学同济医学院附属协和医院
医学伦理委员会（盖章）：

主任/副主任委员签名：[illegible]

日期：2020.2.6

地址：湖北省武汉市解放大道 1277 号 邮编：430000
电话：027--85726685 Fax:0086-27-85726685 E-mial：whunionlunli@126.com
