# Supplementary material for: Study on the promotion of lymphocytes in patients with COVID-19 by broad-spectrum chemokine receptor inhibitor vMIP-II and its Mechanism of signal transmission in vitro
Source: Signal Transduct Target Ther. 2021 Mar 2;6:104. doi: 10.1038/s41392-021-00516-4 (PMC7921284; doi:10.1038/s41392-021-00516-4)
Supplement: Supplementary file 4 — Clinical Approval of vMIP for AIDS from National CDE [file 41392_2021_516_MOESM4_ESM.pdf]

State Food and Drug Administration

Approval documents for clinical research of drugs

Original number： S44030422

Receiving number： X0306933 Batch number：2004L04866

Drug name	recombinant virus macrophage inflammatory protein(vMIP)for injection		
English name / Latin name	recombinant viral macrophage inflammatory protein for injection		
Dosage form	Freeze-dry powder injection	Application matters	New drug
Specifications	0.25 mg/ needle	Registration classification	Therapeutic biological products category 1
Applicant	Jinan University		
Examination and approval conclusion	According to the Drug Administration Law of the people's Republic of China, after examination, this product conforms to the relevant provisions of new drug examination and approval, and agrees to conduct clinical research on this product.<br><br>What needs to be improved during the clinical period:<br><br>Pharmacy: 1. Continuously improve the efficiency and level of the production process (reduce the level of endotoxin), and increase the yield of the product on the basis of ensuring endotoxin control and product quality. 2. To further study and verify the method of determining the activity of passage cells to this product, establish a method for determining the protein content of this product, set up a strict specific activity standard, and perfect this method as a quality standard before reporting for production.<br><br>Toxicology: while clinical research is carried out, long-term toxicity test should be carried out moderately in advance, simulate long-term drug administration as far as possible, pay attention to observe immunotoxicity, antibody production, toxic target organ damage and so on, and design reasonable experiments. Attention should be paid to the general reproductive toxicity and perinatal toxicity during clinical research.<br><br>During the clinical period, attention should be paid to the identification of the function of CD cells and the fluctuation of the proportion of CD cells.<br><br>Efficacy: the efficacy of AIDS treatment drugs should be a stable level of long-term treatment, as important as safety. The current animal experiments suggest that the effect is limited, so it is necessary to conduct an exploratory study on the efficacy during the phase 1 clinical period. For whether the efficacy of this product is time-dependent and viral load-dependent, as well as whether there are other aspects of chemotactic activity in vivo, we can comprehensively consider whether and how to carry out the research according to the results of clinical studies.		
Main delivery	Jinan University		
Carbon Copy	Guangdong Food and Drug Administration, China Institute for the Control of Pharmaceutical and Biological products, Drug Evaluation Center of the State Food and Drug Administration		
Remarks			

22 December 2004

国家食品药品监督管理局

药 物 临 床 研 究 批 件

原始编号：S44030422
受 理 号：X0306933
批 件 号：2004L04866

药物名称	注射用重组病毒巨噬细胞炎性蛋白(vMIP)		
英文名/拉丁名	recombinant viral macrophage inflammatory protein for injection		
剂型	冻干粉针	申请事项	新药
规格	0.25mg/支	注册分类	治疗用生物制品第1类
申请人	暨南大学		
审批结论	根据《中华人民共和国药品管理法》，经审查，本品符合新药审批的有关规定，同意本品进行临床研究。<br>需在临床期间不断完善的内容：<br>药学方面：1、不断提高生产工艺的效率和水平（降低内毒素水平），在确保内毒素控制和产品质量的基础上，增加产品的收率。2、进一步研究和验证传代细胞对本品活性的测定方法，建立本品蛋白含量测定方法，设立严格的比活性标准，并在报生产前完善该方法作为质量标准的检定。<br>毒理方面：在临床研究开展的同时，应有适度提前的长毒试验进行，尽可能模拟长期给药的情况，注意观察免疫毒性、抗体产生、毒性靶器官损伤等，设计合理的试验。临床研究期间应注意观察本品的一般生殖毒性和围产期毒性。<br>在临床期间，注意CD细胞功能的测定，CD细胞比例的波动等。<br>药效方面：艾滋病治疗药物的药效应该是长期治疗水平的稳定，与安全性同等重要。目前的动物试验提示效果有限，因此在1期临床期间必须对药效进行探索性研究。对于本品的药效作用是否存在时间依赖性及病毒载量依赖性，以及在体内是否存在其他方面的趋化活性，可根据临床研究的结果，综合考虑是否以及如何进行研究的问题。		
主送	暨南大学		
抄送	广东省食品药品监督管理局，中国药品生物制品检定所，国家食品药品监督管理局药品审评中心		
备注			

国家食品药品监督管理局
药品注册专用章
2004年12月22日

扫描全能王 创建
